# Benchmarking the methods for predicting base pairs in RNA–RNA interactions

**DOI:** 10.1093/bioinformatics/btaf289

**Published:** 2025-05-06

**Authors:** Mei Lang, Thomas Litfin, Ke Chen, Jian Zhan, Yaoqi Zhou

**Affiliations:** Institute of Systems and Physical Biology, Shenzhen Bay Laboratory, Shenzhen 518107, China; Institute for Biomedicine and Glycomics, Griffith University, Southport, QLD 4222, Australia; Institute of Systems and Physical Biology, Shenzhen Bay Laboratory, Shenzhen 518107, China; Institute of Systems and Physical Biology, Shenzhen Bay Laboratory, Shenzhen 518107, China; Ribopeutic Inc, Futian, Shenzhen, Guangdong 518000, China; Institute of Systems and Physical Biology, Shenzhen Bay Laboratory, Shenzhen 518107, China; Institute for Biomedicine and Glycomics, Griffith University, Southport, QLD 4222, Australia

## Abstract

**Motivation:**

The intricate network of RNA–RNA interactions, crucial for orchestrating essential cellular processes like transcriptional and translational regulations, has been unveiling through high-throughput techniques and computational predictions. As experimental determination of RNA–RNA interactions at the base-pair resolution remains challenging, a timely update for assessing complementary computational tools is necessary, particularly given the recent emergence of deep-learning-based methods.

**Results:**

Here, we employed base pairs derived from three-dimensional RNA complex structures as a gold standard benchmark to assess the performance of 23 different methods ranging from alignment-based methods, free-energy-based minimization to deep-learning techniques. The result indicates that a deep-learning-based method, SPOT-RNA, can be generalized to make accurate zero-shot predictions of RNA–RNA interactions not only between previously unseen RNA structures but also between RNAs without monomeric structures. The finding underscores the potential of deep learning as a robust tool for advancing our understanding of these complex molecular interactions.

**Availability and implementation:**

All data and codes are available at https://github.com/meilanglang/RNA-RNA-Interaction

## Graphical abstract

**Figure:**
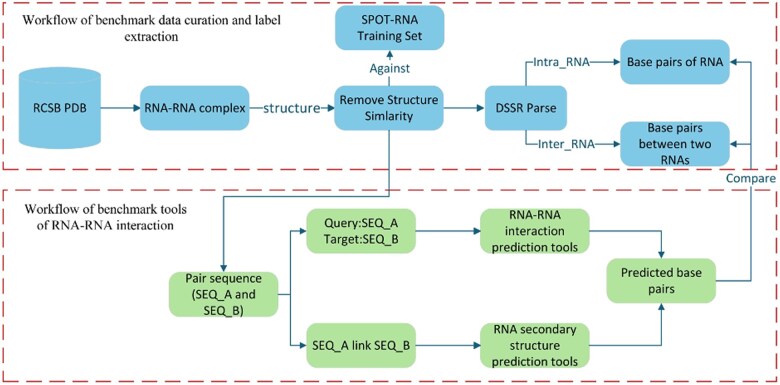

## 1 Introduction

Recent advancements in high-throughput techniques have unveiled a complex network of RNA–RNA interactions (RRIs) critical for governing transcriptional and translational processes. These interactions are pivotal in the biogenesis of various RNA molecules, including mRNAs, rRNA, tRNA, microRNAs, and circRNAs (Gong *et al.* 2018, Dai *et al.* 2020, Singh *et al.* 2022). Large-scale detection of RRIs has been achieved through innovative approaches that combine cross-linking techniques with high-throughput sequencing. Notably, techniques such as PARIS (Lu *et al.* 2016), SPLASH (Aw *et al.* 2016), LIGR-seq (Sharma *et al.* 2016), and COMRADES (Ziv *et al.* 2018) have employed psoralen or its derivatives, as well as formaldehyde in the case of RIC-Seq (Cai *et al.* 2020), for cross-linking. While these methods hold promise, they are not without limitations stemming from probe biases and ligation efficiencies (Gong *et al.* 2018, Dai *et al.* 2020, Singh *et al.* 2022). For example, KARR-seq, PARIS, and RIC-seq can only achieve about 55% true positive rate at 20% false positive rate for human 18S rRNA (Wu *et al.* 2024). Furthermore, many of these high-throughput techniques have yet to achieve the single nucleotide resolution.

Attaining the nucleotide-level resolution in RNA structures has historically relied on traditional structure-determination methods such as X-ray crystallography, Nuclear Magnetic Resonance (NMR), and Cryo-electron microscopy. Yet, compared to proteins, determining RNA structures presents formidable challenges due to the unique physiochemical properties of nucleotides and the inherent fragility of RNA structures (Xu *et al.* 2022). This is reflected from the fact that only a meager 3% of structures in the Protein Data Bank contain RNAs, with even fewer dedicated to RNA–RNA complexes (681 as of 16 March 2023, before redundancy removal) (Burley *et al.* 2021). This stark contrast becomes even more pronounced when considering the extensive collection of more than 31 million noncoding RNA sequences catalogued in the RNAcentral database (RNAcentral Consortium 2021). Given the cost and challenges associated with experimental approaches, there is an imperative need for development of complementary computational prediction techniques.

The existing methods for predicting RNA–RNA interactions (RRIs) can be broadly classified into alignment-based, free-energy-based, and homology modeling approaches (Lai and Meyer 2016, Umu and Gardner 2017). Alignment-based techniques, such as GUUGle (Gerlach and Giegerich 2006) and RIsearch (Wenzel *et al.* 2012), focus on inter-RNA base pairs while overlooking potential intra-RNA interactions. Free-energy-based methods can be categorized into those considering only intermolecular interactions for expediency [such as RNAhybrid (Krüger and Rehmsmeier 2006), RNAduplex (Lorenz *et al.* 2011), RNAplex-c (Tafer and Hofacker 2008), and DuplexFold (Reuter and Mathews 2010)], those factoring in intramolecular interactions based on solvent accessibility [such as RNAup (Mückstein *et al.* 2006), IntaRNA (Busch *et al.* 2008), RNAplex-a (Tafer *et al.* 2011), and AccessFold (DiChiacchio *et al.* 2016)], and those accommodating both intra- and inter-molecular base pairs through sequence concatenation [such as PAIRFOLD (Andronescu *et al.* 2005), RNAcofold (Bernhart *et al.* 2006) and biFold (Reuter and Mathews 2010)] or without restrictions (such as RactIP (Kato *et al.* 2010)). Homology-based techniques, exemplified by TargetRNA2 (Kery *et al.* 2014), CopraRNA (Wright *et al.* 2013), RNAaliduplex (Lorenz *et al.* 2011) and PETcofold (Seemann *et al.* 2011), utilize evolutionary information to infer binding.

Presently, the above “de novo” RRI prediction methods predominantly rely on free-energy-based approaches. Like energy-based methods in intra-RNA secondary structure prediction (Zhao *et al.* 2018), these methods are not only limited by their inherent inaccuracy in energy or scoring functions, but also typically ignore pseudoknotted base pairs except IntaRNA2 (Busch *et al.* 2008) due to algorithmic complexity and intensive computational requirements. In RNA secondary structure, a pseudoknot is a type of RNA structure that occurs when a single-stranded region of RNA folds back and forms base pairs with another, non-adjacent single-stranded region, resulting in a “knot-like” structure (Staple and Butcher 2005). Simple pseudoknot is defined when there is a relationship between two base pairs (i, j) and (k, l), where: i < k < j < l, i.e. the two base pairs cross over each other in the sequence. Kissing hairpins (also known as kissing-loops or kissing stem-loops), playing a crucial role in RNA–RNA interactions structure and function (You and Rice 2008, Ganser and Al-Hashimi 2016, Bose *et al.* 2024), may become pseudoknots when two chains are concatenated. Thus, those concatenation-based methods that do not account for pseudoknots prior to concatenation may well be inherently at disadvantage for predicting RRIs between structured RNAs.

Recently, advancements have seen the emergence of deep-learning-based methods, starting with SPOT-RNA (Singh *et al.* 2019), which achieved the first end-to-end prediction of intra-RNA base pairs that account for base pairs stabilized for tertiary interactions, including pseudoknots, lone base pairs, base-triplets, and non-canonical base pairs. To further enhance prediction accuracy, SPOT-RNA2 (Singh *et al.* 2021) was further developed to integrate evolutionary profiles and mutational coupling data generated by RNAcmap (Zhang *et al.* 2021). Subsequent development of other deep-learning techniques includes mxfold2 (Sato *et al.* 2021), UFold (Fu *et al.* 2022), and 2dRNA (Mao *et al.* 2022). However, not all these deep-learning techniques account for pseudoknots.

In this study, we conducted a comprehensive benchmark of various methods for predicting inter-RNA interactions. Our evaluation encompassed traditional energy-based techniques and newly developed deep-learning models based on simple sequence concatenation. To ensure a rigorous assessment, we employed base pairs derived from experimentally-determined RNA–RNA complex structures and eliminated monomer structures employed for training SPOT-RNA and SPOT-RNA2 through a strict structural similarity cut-off [TM-score (Gong *et al.* 2019) <0.3], in addition to sequence similarity cut off by CD-HIT. This challenging set of the complex of unseen structures revealed significant improvements of SPOT-RNA’s performance over the other 22 methods evaluated, underscoring the transferability of deep learning from intra-RNA to inter-RNA interaction prediction. The performance by SPOT-RNA is robust against different types of RNA complexes. Further tests on training-family-excluded test sets confirm the robustness of SPOT-RNA in hetero-dimeric complexes of RNAs, in particular.

## 2 Materials and methods

### 2.1 Benchmark datasets

We retrieved all RNA structures from the Protein Data Bank (PDB) in March 2023 (Burley *et al.* 2021) and specifically selected structures featuring two RNA chains with a minimum of five inter-RNA base pairs. The identification of base pairs was carried out using Dissecting the Spatial Structure of RNA (DSSR) (Lu *et al.* 2015). For base-pair prediction in complex structures, base pairs that form interactions between two RNAs in the complex are positive samples, whereas unpaired bases between them are negative samples. To eliminate redundancy, we applied CD-HIT-EST (Li and Godzik 2006): an RNA complex is excluded from the dataset if individual RNA sequences in the RNA complex demonstrate sequence similarity (over 80% identity) to any RNA sequences in other complexes of the same dataset. This initial step resulted in 155 unique RNA–RNA interaction (RRI) pairs.

To ensure stringency, we further filtered out RRI pairs that exhibited single-chain structural similarities with any RNAs in the SPOT-RNA training set, defined by TM-score ≥0.3 using RNA-align (Zheng *et al.* 2019) with the length of the query sequence for normalization. This rigorous process yielded a final benchmark set comprising 64 unique RRI pairs. The PDB IDs of the benchmark set can be found in Supplementary Table S1. To further build a dataset excluding RNA families employed in training/validating SPOT-RNA, we identified 14 complexes out of 64 test complexes whose RNAs do not belong to any RFAM families with RNAs contained in training/validation TR0/VL0 [bpRNA (Danaee *et al.* 2018)] and TR1/VL1 (RNAs with 3D structures) sets of SPOT-RNA.

To expand this dataset, we re-downloaded all RNA complexes on 28 March 2024 from the protein data bank and processed by DSSR. Potential complexes were extracted from asymmetric units and biological assemblies and then filtered such that only structures with six or more intermolecular and three or more intramolecular base pairs (canonical and non-canonical) per subunit were retained in the dataset. Any complex with a subunit of length >400 nucleotides (almost exclusively rRNA and snRNA) or containing only subunits with length less than 25 nucleotides were removed from the dataset. Remaining RNA were filtered against the SPOT-RNA training/validation sets (TR0/VL0/TR1/VL1) using blastn (-task blastn-short) with an e-value cutoff of 0.001 and using RNA-align structure alignment program with a cutoff of 0.45. Identical duplicates were removed by selecting structures with the largest number of base-pairing interactions. Two RNA (7r6q—rRNA and 6ltp—crRNA) were also removed after manual inspection. Finally, we found that except for three of the new complexes, all other complexes are homo-oligomers and among the original complexes, only two of them are homo-oligomeric. Therefore, we integrated these two datasets, resulting in a homo-dimer data set containing 13 complexes and a hetero-dimer data set containing 15 complexes.

### 2.2 Performance evaluation

We evaluated performance using common metrics: Recall (sensitivity), Precision, and the F1-score. PPV/Precision is TP/(TP+FP), Sensitivity/Recall is TP/(TP+FN), and the F1-score is 2(Recall*Precision)/(Recall + Precision). Here, TP, FP, and FN are true positives, false positives, and false negatives, respectively. We also calculated Matthew’s correlation coefficient (MCC) to provide a balanced measure as below:


(1) $$\mathrm{MCC}=\frac{TP\times TN-FP\times FN}{\sqrt{\left(TP+FP)(TP+FN)(TN+FP)(TN+FN\right)}}$$

where TN denotes true negatives. MCC considers true negatives (TN) and measures the correlation between expected and observed classes. It ranges from 0 (no correlation) to 1 (highest correlation).

### 2.3 Methods for interaction prediction

We summarized the methods compared in Table 1, with detailed settings for each method provided in the Supplementary Method description in the Supplementary Data. As per Lai and Meyer (2016), we categorized the algorithms into four types: (i) “Interaction only” methods predict intermolecular hybridization, ignoring RNA secondary structures; (ii) “Accessibility” methods consider RNA secondary structures using a partition function for unpaired probability; (iii) “Concatenation” algorithms treat two input sequences as a single chain and predict a joint secondary structure; and (iv) “Complex joint” methods also predict joint secondary structures but without concatenating the input sequences.

**Table 1.** RRI interaction tools employed in this study for comparison, listed according to the year of publication along with their categories, the use of evolution information (MSA), the algorithm, the capability of predicting intra-RNA base pairs, and the ability to predict pseudoknots.

Methods	Ref	Year	Category^a^	MSA^b^	Algorithms^c^	Intra-RNA base pairs^d^	Pseudoknots^e^
RNAfoldc^f^	(Mathews *et al.* 2004)	2004	Concatenation	No	MFE	Yes	No
PairFold	(Andronescu *et al.* 2005)	2005	Concatenation	No	MFE	Yes	No
RNAup	(Mückstein *et al.* 2006)	2006	Accessibility	No	MFE	No	No
GUUGle	(Gerlach and Giegerich 2006)	2006	Interaction only	No	MFE	No	No
RNAcoFold	(Bernhart *et al.* 2006)	2006	Concatenation	No	partition + MFE	Yes	No
NUPACK	(Dirks *et al.* 2007)	2007	Concatenation	No	MFE	No	No
RNAplex-c	(Tafer and Hofacker 2008)	2008	Interaction only	No	MFE	No	No
RNAplex-a	(Tafer and Hofacker 2008)	2008	Accessibility	No	MFE	No	No
RNAplex-cA	(Tafer and Hofacker 2008)	2008	Interaction only	Yes	MFE	No	No
bifold	(Reuter and Mathews 2010)	2010	Complex joint	No	MFE	No	No
DuplexFold	(Reuter and Mathews 2010)	2010	Interaction only	No	MFE	No	No
PETcoFold	(Seemann *et al.* 2011)	2011	Complex joint	Yes	MFE	Yes	No
RNAduplex	(Lorenz *et al.* 2011)	2011	Interaction only	No	MFE	No	No
RNAaliduplex	(Lorenz *et al.* 2011)	2011	Interaction only	Yes	MFE	No	No
RNAmultifold	(Lorenz *et al.* 2011)	2011	Concatenation	No	MFE	Yes	No
RIsearch	(Wenzel *et al.* 2012)	2012	Interaction only	No	MFE	No	No
AccessFold	(DiChiacchio *et al.* 2016)	2016	Concatenation	No	MFE	No	No
IntaRNA2.0	(Mann *et al.* 2017)	2017	Accessibility	No	MFE + partition	No	Yes
SPOT-RNAc^f^	(Singh *et al.* 2019)	2019	Concatenation	No	DL	Yes	Yes
SPOT-RNA2c^f^	(Singh *et al.* 2021)	2021	Concatenation	Yes	DL	Yes	Yes
MXfold2c^f^	(Sato *et al.* 2021)	2021	Concatenation	No	DL	Yes	No
UFoldc^f^	(Fu *et al.* 2022)	2022	Concatenation	No	DL	Yes	Yes
EternaFoldc^f^	(Wayment-Steele *et al.* 2022)	2022	Concatenation	No	MFE	Yes	No

a Category: the broad category of the method (see text for more details).

b MSA—indicate whether it takes multiple sequence alignment as input.

c Algorithm: MFE: minimum free energy, Partition; partition function, DL: deep learning.

d Intra-RNA base pairs—whether the output of a software also contains base-pairing information for intramolecular interactions.

e Pseudoknots—indicates whether it can predict pseudoknots.

f c—indicates concatenation of two chains. For example, SPOT-RNAc and SPOT-RNA2c denotes SPOT-RNA and SPOT-RNA2 with sequence concatenation, respectively, to distinguish from the methods dedicated to individual chains (SPOT-RNA and SPOT-RNA2, respectively).

We employed concatenation for comparing recent deep-learning methods with traditional free-energy-based methods. When dealing with concatenated chains, we made predictions for both sequence orders (AB and BA) and reported the average if probabilities were predicted. For those methods providing a two-state prediction, we considered the union of base pairs predicted for both sequence orders as a positive prediction (i.e. a positive prediction from either AB or BA concatenation will be considered as positive).

We experimented with and without a three-nucleotide linker (AAA, UUU, CCC, or GGG) and found that direct concatenation without any linker yielded slightly better results, although not statistically significant compared to AAA/CCC linkers (Supplementary Table S2). Therefore, we report results based on direct concatenation without any linkers.

### 2.4 Homology search

For the methods that employed homologous sequences without chain linking such as RNAplex-cA, PETcoFold, RNAaliduplex, and SPOT-RNA2, we searched the homologs using the single chain by RNAcmap3 (Chen *et al.* 2024) which is based on MARS database (a comprehensive database by including the RNAcentral, MG-RAST, GWH, MGnify and NCBI’s nucleotide databases). For SPOT-RNA2c using a link chain, we searched the homologs with the linked chain by RNAcmap3. The median number of homologous sequences found is 1467 for single chains and 940 for linked chains.

### 2.5 Identification of pseudoknots

We employed bpRNA (Danaee *et al.* 2018) to identify pseudoknots. By inputting the base pairs for a given RNA (either from prediction or from PDB structures), bpRNA provides an easily-interpretable description of all loops, stems, and pseudoknots. We use bpRNA on monomer structures as well as concatenated structures (predicted or actual PDB structures) from two chains in complex with each other. The pseudoknot base pairs between two RNA chains from either AB or BA linkage are defined as intermolecular pseudoknots.

## 3 Results

### 3.1 Method comparison on inter-RNA base pair prediction

**Figure 1.**
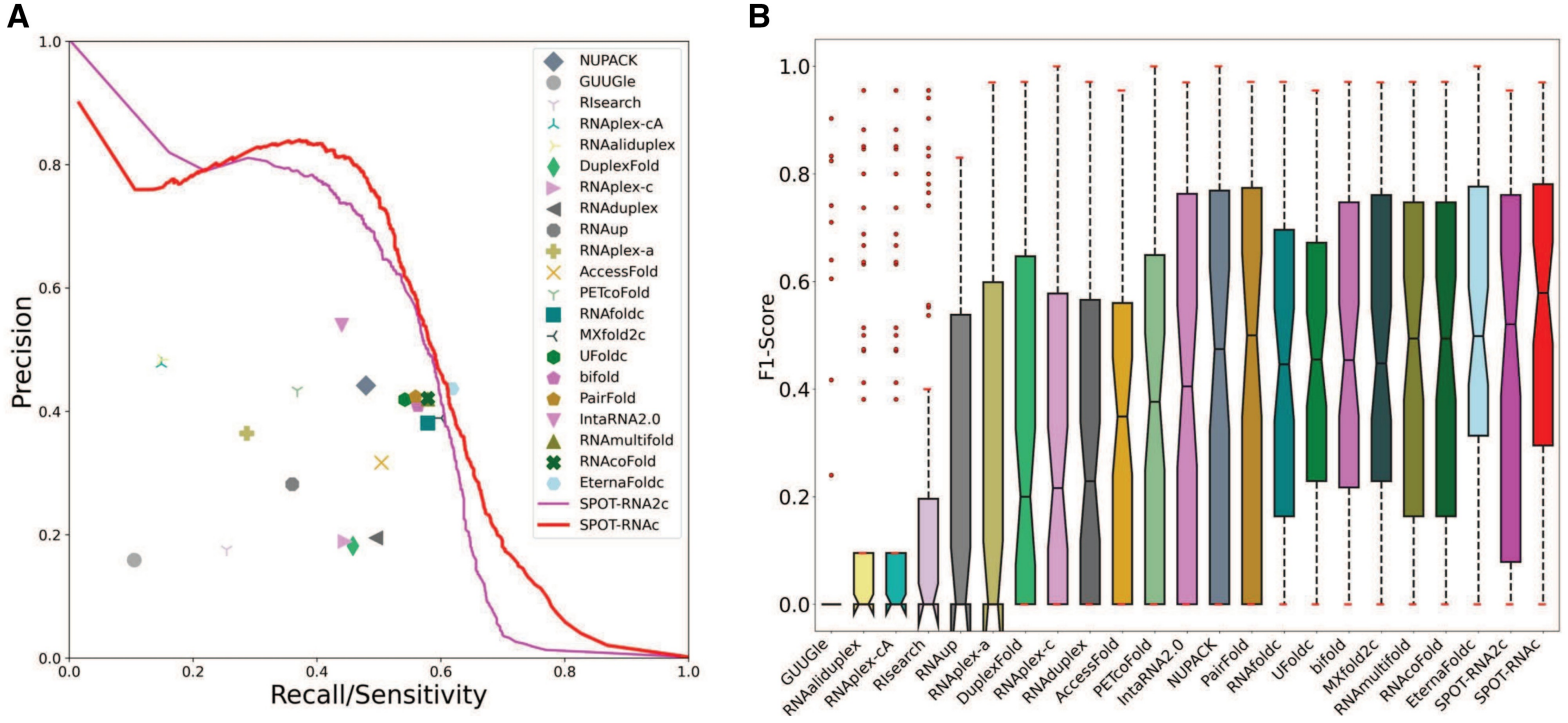
Performance comparison of 23 methods for inter-RNA base-pair prediction on the 64 complexes of RNA structures unseen by SPOT-RNA and SPOT-RNA2. All structures in the test set have the structural similarity score TM-score < 0.3 compared to the monomeric structures used in training and validating SPOT-RNA and SPOT-RNA2 methods. (A) Precision-recall curves (for those methods with probabilistic outputs) or points given by 23 methods (B) Distribution of F1-scores for inter-RNA base-pair prediction for individual RNA pairs by the same 23 methods. Each boxplot shows the median, 25th, and 75th percentiles, with outliers represented by “•”. SPOT-RNAc exhibits the best performance for both overall and individual measures of F1-scores.

In this study, we compared 23 different RRI predictors on a benchmark set of 64 RNA–RNA pairs after excluding all monomer structures remotely similar to the RNA structures employed in the training and validation sets for SPOT-RNA and SPOT-RNA2. We evaluated their performance in untrained inter-RNA base pair prediction through precision/recall curves and F1-score distributions, as shown in Fig. 1. The performance metrics, including overall F1-score, MCC values, and the median F1-score and standard deviation of individual RNA pairs, are also summarized in Table 2. Predictors with probabilistic outputs are represented by precision-recall (PR) curves, while others are represented as single points. For all methods with chain concatenation for predicting RNA–RNA interactions, a low case “c” is appended to the method name. They are RNAfoldc, UFoldc, MXfold2c, EternaFoldc, SPOT-RNAc, and SPOT-RNA2c. No linker was employed because adding a 3-nucleotide link did not lead to performance improvement (See Section 2).

**Table 2.** Performance comparison of 23 predictors of inter-RNA base pairs on 64 complexes of RNA structures unseen by SPOT-RNA and SPOT-RNA2^a^.

Methods	PPV/Precision	Sensitivity/Recall	Overall F1-score	MCC	Individual F1-score median ± Std	*P*-value
GUUGle	0.159	0.105	0.126	0.128	0.000 ± 0.241	8.84e-15
RIsearch	0.176	0.254	0.208	0.210	0.000 ± 0.319	1.87e-12
RNAplex-cA*	0.477	0.149	0.227	0.266	0.000 ± 0.296	2.38e-11
RNAaliduplex	0.484	0.149	0.228	0.268	0.000 ± 0.296	2.38e-11
DuplexFold	0.182	0.458	0.260	0.287	0.200 ± 0.344	9.20e-8
RNAplex-c	0.189	0.445	0.265	0.289	0.216 ± 0.343	1.49e-8
RNAduplex	0.195	0.495	0.280	0.309	0.229 ± 0.329	1.51e-8
RNAup	0.282	0.360	0.316	0.317	0.000 ± 0.290	1.94e-12
RNAplex-a	0.364	0.287	0.321	0.322	0.000 ± 0.331	2.39e-9
AccessFold	0.317	0.504	0.389	0.398	0.349 ± 0.315	1.00e-5
PETcoFold*	0.434	0.368	0.398	0.399	0.377 ± 0.352	1.42e-4
NUPACK	0.442	0.479	0.460	0.459	0.474 ± 0.355	2.89e-3
RNAfoldc	0.381	0.579	0.460	0.469	0.446 ± 0.318	0.011
MXfold2c	0.389	0.601	0.472	0.482	0.448 ± 0.320	0.008
Ufoldc	0.419	0.542	0.473	0.476	0.455 ± 0.302	4.04e-3
Bifold	0.409	0.563	0.474	0.479	0.454 ± 0.325	0.022
PairFold	0.424	0.559	0.482	0.486	0.500 ± 0.339	0.012
IntaRNA2.0	0.540	0.440	0.485	0.486	0.405 ± 0.377	1.55e-4
RNAmultifold	0.420	0.579	0.487	0.492	0.494 ± 0.325	0.025
RNAcoFold	0.421	0.579	0.488	0.493	0.494 ± 0.325	0.025
EternaFoldc	0.437	**0.618**	0.512	0.519	0.498 ± 0.314	0.134
SPOT-RNA2c*	0.569	0.537	0.553	0.552	0.529 ± 0.329	0.020
SPOT-RNAc	**0.606**	0.548	**0.576**	**0.576**	**0.579 **±** **0.323	

Note: The overall F1-score is harmonic mean of precision and recall for all RNA pairs. PPV denotes positive predictive value. MCC denotes Matthews’ correlation coefficient. The star * denotes the use of evolution information. Methods with an ending of “c” indicate the use of chain concatenation for RNA–RNA interaction prediction. Median F1 means the median F1 value of single RNA. The *P*-value of a given method was computed by against the result from SPOT-RNAc and was calculated using Student’s t-test. Bold values highlight the best performance values.

a All single-chain structures in the test set have the structural similarity score TM-score < 0.3 compared to the monomeric structures used in training and validating SPOT-RNA and SPOT-RNA2 methods.

As shown in Fig. 1A, SPOT-RNA2c is the most accurate predictor at low sensitivity (<0.2). However, the overall PR curve given by SPOT-RNAc has the best performance. We can also measure the performance by the overall F1-score for all RNA pairs and the median F1-score for individual RNA pairs. The thresholds for determining F1-scores of SPOT-RNAc and SPOT-RNA2c in the test set were set according to the thresholds for producing the highest F1-scores in the validation dataset for SPOT-RNAc and SPOT-RNA2c, respectively. Table 2 confirmed the result from the PR curve that SPOT-RNAc achieved the best overall performance with an overall F1-score of 0.576, outperforming SPOT-RNA2c (the overall F1-score of 0.553) and EternaFoldc (F1-score of 0.512). SPOT-RNAc improves over SPOT-RNA2c by more than 4% and outperforms other methods by over 13%, a pattern similarly observed in MCC values (Table 2). Figure 1B presents the distribution of F1-scores for individual RNA pairs, including median, 25th, and 75th percentiles. SPOT-RNAc continues to achieve the best performance with the highest median F1-score of 0.579, outperforming the next best SPOT-RNA2c (median F1-score of 0.529) with a 9% improvement. The improvement of SPOT-RNAc over all methods are statistically significant except EternaFold (Table 2) according to student’s t-test.

As many methods do not predict pseudoknots, it is of interest to know if the performance of SPOT-RNA was benefited from its inclusion of pseudoknot prediction. We employed bpRNA to analyze the existence of pseudoknots in our dataset and identified pseudoknots only in 8 out of 64 RNA interaction pairs in either A–B or B–A linkage. In Supplementary Table S3, we excluded pseudoknots from both the ground-truth labels and predicted base pairs. As the table shows, SPOT-RNAc remains the best according to F1-score and MCC values.

**Figure 2.**
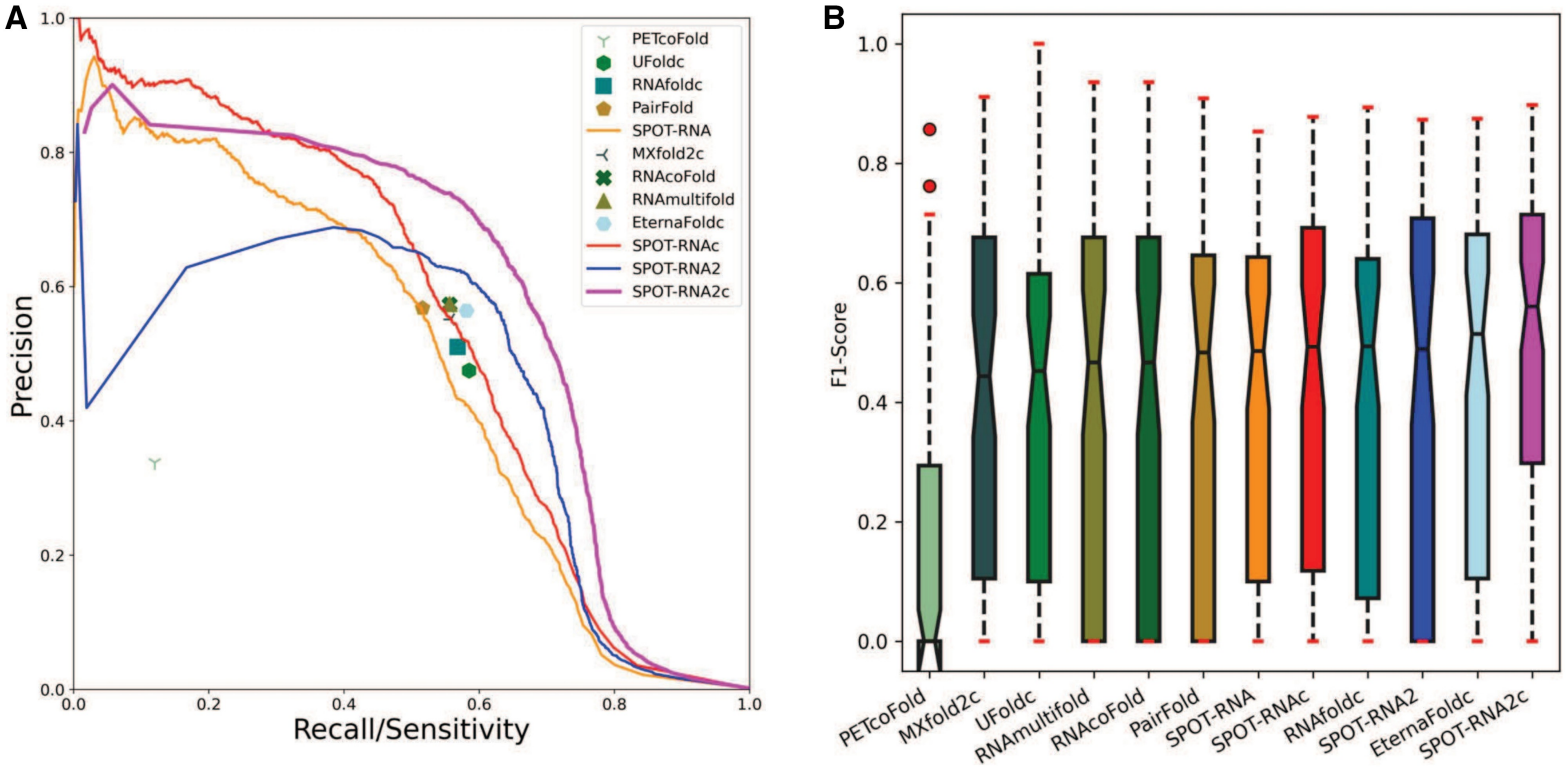
Performance comparison of intra-RNA base pair prediction by 12 methods (A) Precision-recall curves and points illustrate the performance rankings. (B) A distribution of F1-scores for intra-RNA base-pair prediction. SPOT-RNA and SPOT-RNAc represent intramolecular base-pair prediction as single and concatenated chains, respectively. Evolution-based SPOT-RNA2c (or SPOT-RNA2) outperforms SPOT-RNAc (or SPOT-RNA) for intra-RNA base pairs.

It’s important to assess how these methods perform on intra-molecular interactions, although not all RRI methods offer predictions for such interactions. We remove these RNAs without intra-base-pairing structures. This leads to 77 RNA chains. Figure 2A compares the PR curves or PR points given by 12 methods. Interestingly, PR curves indicate that SPOT-RNA2c now has the best performance. The overall performance according to overall F1-scores given by SPOT-RNA2c (Table 3) is the highest, surpassing the next best methods (SPOT-RNA2 without chain concatenation) by a 7% improvement in F1-score, and the third best (SPOT-RNAc) by 10%. This trend is consistent with MCC values.

**Table 3.** Performance comparison of 12 predictors for predicting intra-RNA base pairs on 77 unseen RNA structures that have intra secondary structure in 64 RNA–RNA complex structures.

Methods	PPV/Precision	Sensitivity/Recall	Overall F1-score	MCC	Individual F1-score median ± Std	*P*-value
PETcoFold*	0.338	0.120	0.177	0.200	0.000 ± 0.214	8.41e-15
UFoldc	0.475	0.585	0.524	0.526	0.452 ± 0.278	4.82e-07
RNAfoldc	0.510	0.568	0.537	0.537	0.494 ± 0.284	6.05e-06
PairFold	0.568	0.516	0.541	0.540	0.483 ± 0.306	7.64e-05
SPOT-RNA	0.589	0.505	0.544	0.544	0.486 ± 0.281	1.43e-06
MXfold2c	0.551	0.558	0.554	0.553	0.444 ± 0.290	1.26e-04
RNAcoFold	0.573	0.556	0.564	0.563	0.467 ± 0.303	8.84e-04
RNAmultifold	0.574	0.556	0.565	0.563	0.467 ± 0.303	8.84e-04
EternaFoldc	0.564	0.581	0.572	0.571	0.514 ± 0.291	5.62e-04
SPOT-RNAc	0.663	0.513	0.578	0.582	0.493 ± 0.292	7.62e-04
SPOT-RNA2*	0.573	0.622	0.596	0.596	0.489 ± 0.308	0.007
SPOT-RNA2c*	**0.614**	**0.658**	**0.635**	**0.635**	**0.560 **±** **0.292	

Note: The overall F1-score is harmonic mean of precision and recall for all RNA pairs. PPV denotes positive predictive value. MCC denotes Matthew’s correlation coefficient. The star * indicates the use of evolution information. Std means standard deviation. Median F1 means the median F1 value of individual RNA chains. The *P*-value of a given method is calculated by against the result of SPOT-RNA2c and was calculated using Student’s t-test. Bold values highlight the best performance values.

Figure 2B further examines the distribution of F1-scores for individual RNAs. SPOT-RNA2c continues to lead with the highest median F1-score at 0.560, while EternaFoldc follows closely with the second-best median F1-score of 0.514. The differences in F1-score distributions between SPOT-RNA2c and other methods are all statistically significant, with a *P*-value of 5.62 × 10^–4^ when comparing SPOT-RNA2c to the second-best EternaFoldc (Table 3).

**Figure 3.**
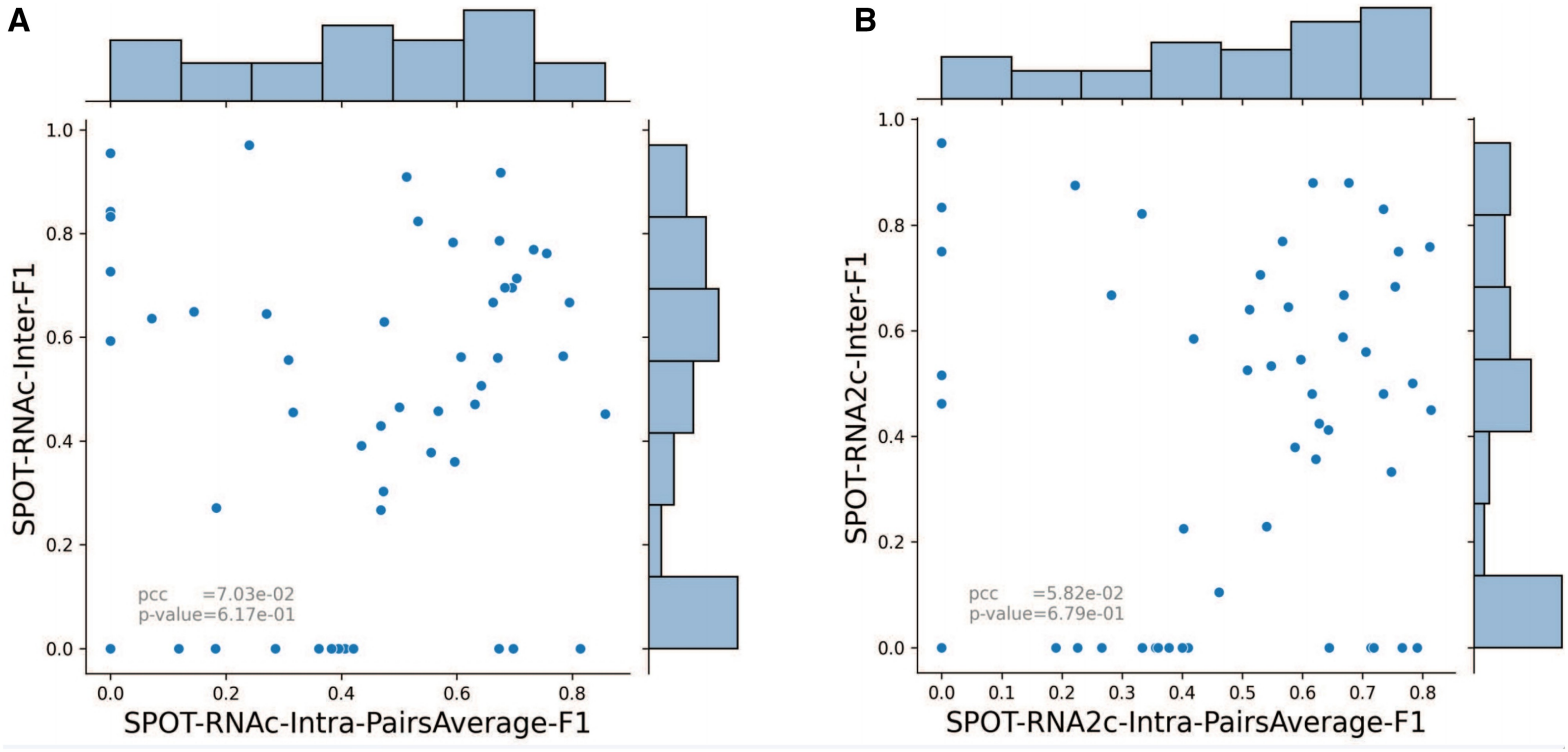
No correlation between inter-RNA F1-scores and intra-RNA F1-scores. (A) Inter-RNA F1-scores versus the average intra-RNA F1-scores of SPOT-RNAc for 53 RNA complex structures with intra-RNA base pairs for both chains. (B) Inter-RNA F1-scores versus intra-RNA F1-scores of SPOT-RNA2c for 53 RNA complex structures.

Intuitively, a better intra-RNA base-pairing prediction should lead to a better inter-RNA base-pairing prediction. However, although SPOT-RNA2c has the best performance for intra-RNA interaction prediction, it is SPOT-RNAc with the best performance for inter-RNA interaction prediction. If we remove these RNA RRI pairs of which both chains do not have intra-base-pairing structures, this leads to 53 RRI pairs. Figure 3A compares intermolecular F1-scores for individual RNA pairs from SPOT-RNAc with the average intramolecular F1-scores. No correlation was found. Similar uncorrelated intra- and inter-RNA F1-scores are observed for SPOT-RNA2c (Fig. 3B). This suggests that the evolution information contained in SPOT-RNA2c did contain the co-evolution information for predicting intra-RNA, but not inter-RNA interactions.

We also assessed the impact of sequence concatenation on the intra-RNA base-pair prediction (i.e. SPOT-RNA versus SPOT-RNAc, SPOT-RNA2 versus SPOT-RNA2c). Table 3 shows that SPOT-RNAc/SPOT-RNA2c are better than SPOT-RNA/SPOT-RNA2 based on either overall F1-score or the median of individual F1-scores. In both cases, the difference is statistically significant with a *P*-value of 0.0006 between SPOT-RNA and SPOT-RNAc and a *P*-value of 0.007 between SPOT-RNA2 and SPOT-RNA2c. This indicates that knowing the binding partner improves the intra-RNA base-pair prediction. For example, in the complex structure between U2 snRNA and U6 snRNA (Chain A and Chain C of PDB ID 6QDV), SPOT-RNAc (with chain connection) improved over SPOT-RNA (without chain connection) with an F-score of 0.775 over 0.706 for U2 snRNA and 0.480 over 0.323 for U6 snRNA, respectively.

**Figure 4.**
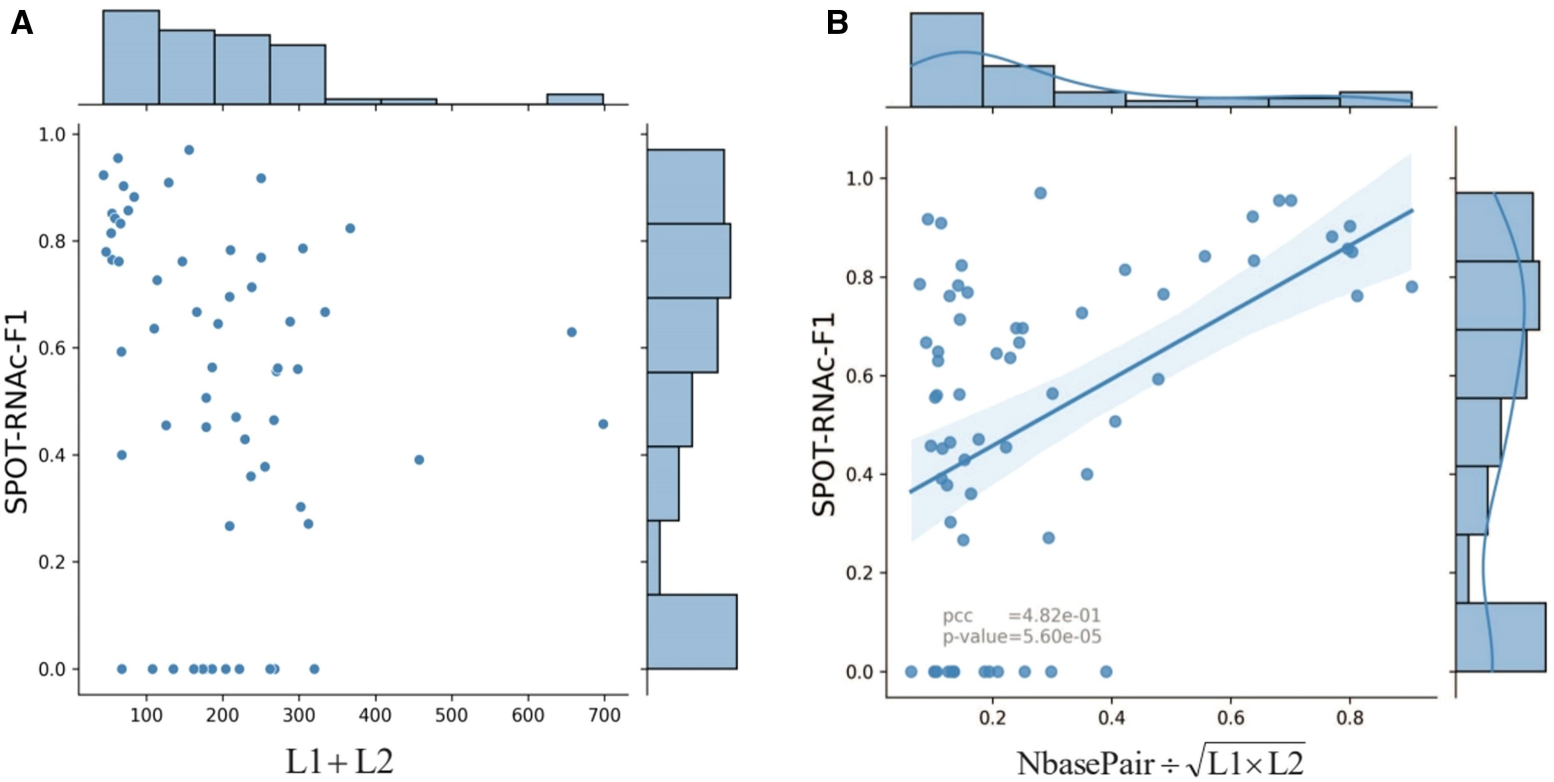
Relationship between inter-RNA base pairing prediction and sequence/interaction characteristics. (A) The inter-RNA F1-scores for individual RNA–RNA complexes from SPOT-RNAc plotted against the sum of sequence lengths (L1 + L2). (B) The inter-RNA F1-scores for individual RNA–RNA complexes from SPOT-RNAc plotted against the normalized number of inter-RNA base pairs (the number of true inter-RNA base pairs divided by the $\sqrt{L1\times L2}$). The performance does not correlate with sequence length but is related to the normalized number of inter-RNA base pairs.

We observed that some inter-RNA (and intra-RNA) interactions were predicted with F1-score of 0 as shown in Fig. 3A and B. To understand the reason behind these poor predictions, we examined F1-scores given by SPOT-RNAc as a function of sequence length (L1 + L2) in Fig. 4A. No obvious correlation was found. However, when we plotted F1-scores against the number of true inter-RNA base pairs divided by the square root of (L1 * L2), a clear and strong correlation emerged with a Pearson’s correlation coefficient (PCC) of 0.482 (Fig. 4B). Thus, poor predictions, including those with F1-scores of 0, can be attributed to the scarcity of inter-RNA contacts relative to the sequence lengths. This observation holds true for intra-RNA base-pair prediction as well: intra-RNA interactions with F1-scores of 0 also involve very few intra-RNA base pairs (<5).

One question is whether the method performance depends on RNA types. We classified 64 complexes into rRNA-containing complexes (only 8), snRNA/snoRNA/mRNA-containing complexes (40), and RNA complexes lacking intra-RNA interactions (16). SPOT-RNAc is consistently the best in each category as shown Supplementary Table S4 for the overall F1-score and MCC values. It is of note that even for RNA complexes lacking intra-RNA interactions (i.e. these extreme cases of unstructured RNAs are not employed for training in neither SPOT-RNA, nor SPOT-RNA2), SPOT-RNAc continues to provide the best overall F1-score and MCC values, although its performance for averaging over individual complexes is statistically similar to the those of some energy-based techniques. The consistency of SPOT-RNAc performance among different RNA types demonstrates that deep learning, even with a limited number of 3D structures for training, can yield generalizable models for inter-RNA base-pair prediction.

Despite the attempt to remove structural homologs from training by structural alignment, some RNA families in training sets (rRNA and spliceosome complexes) were still included in the above 64-complex test sets because RNAs in the same family do not necessarily have an identical structure. We further examined the effect of excluding-training families in intra-base-pair prediction by establishing new datasets. They were obtained by removing training/validation families from 64 test complexes and locating additional new RNA complexes (up to 28 February 2024) (See Section 2). These two datasets are mixed and separated into 15 hetero-dimeric complexes and 13 homo-dimeric complexes. For 15 hetero-dimer complexes, although the difference between SPOT-RNAc and some methods are statistically insignificant, SPOT-RNAc remains the best performance among all the methods compared for the overall performance (Table 4), whereas SPOT-RNA2 remains the best for intra-RNA base-pair prediction (Supplementary Table S5) with an overall F1-score of 0.589, followed by RNAmultifold (0.586) and RNAcoFold (0.586). If ranked by median F1-score, SPOT-RNA2c remains the best for intra-RNA interaction prediction. Most did poorly with a median F1-score of 0 except SPOT-RNA2c, due to low number of intra-RNA contacts for this set of the data. All also did very poorly with median F1-score of 0 for inter-RNA base-pair prediction in the 13-homo-dimer set (Supplementary Table S6), whereas SPOT-RNA2 is the best performance with an overall F1-score of 0.724, followed by SPOT-RNA (0.555) and EternaFoldc (0.541) for intra-RNA base-pair prediction (Supplementary Table S7).

**Table 4.** Performance comparison of all predictors on 15 hetero-dimer complexes of inter-RNA base pairs that are not in RNA families containing in TR0/VL0 (bpRNA) and TR1/VL1 for training SPOT-RNA and SPOT-RNA2.

Methods	PPV/Precision	Sensitivity/Recall	Overall F1-score	MCC	Individual F1-score median ± Std	*P*-value
GUUGle	0.433	0.318	0.367	0.367	0.000 ± 0.355	1.02e-4
RNAaliduplex	0.647	0.331	0.438	0.460	0.381 ± 0.359	6.00e-4
RNAplex-cA*	0.647	0.331	0.438	0.460	0.381 ± 0.359	6.00e-4
RIsearch	0.472	0.744	0.578	0.589	0.781 ± 0.347	0.039
RNAup	0.464	0.764	0.577	0.592	0.615 ± 0.227	2.18e-6
RNAduplex	0.469	0.807	0.593	0.611	0.846 ± 0.301	0.170
RNAplex-a	0.676	0.561	0.613	0.613	0.652 ± 0.271	1.87e-3
PETcoFold*	0.684	0.561	0.616	0.617	0.638 ± 0.304	0.010
DuplexFold	0.559	0.780	0.651	0.657	0.846 ± 0.313	0.212
RNAplex-c	0.572	0.780	0.660	0.665	0.846 ± 0.313	0.212
PairFold	0.634	0.807	0.710	0.713	0.846 ± 0.283	0.222
UFoldc	0.659	0.793	0.720	0.721	0.833 ± 0.274	0.166
bifold	0.684	0.793	0.734	0.734	0.846 ± 0.278	0.235
RNAfoldc	0.672	0.820	0.739	0.740	0.833 ± 0.273	0.134
NUPACK	0.698	0.797	0.744	0.744	0.846 ± 0.271	0.374
EternaFoldc	0.684	0.816	0.744	0.745	0.833 ± 0.269	0.125
SPOT-RNA2c*	0.710	0.787	0.747	0.745	0.821 ± 0.291	0.066
RNAmultifold	0.702	0.797	0.746	0.746	0.846 ± 0.276	0.131
RNAcoFold	0.702	0.797	0.746	0.746	0.846 ± 0.276	0.131
2.0	0.713	0.800	0.754	0.754	0.846 ± 0.272	0.419
MXfold2c	0.684	**0.839**	0.754	0.756	0.833 ± 0.210	0.627
AccessFold	0.696	0.826	0.755	0.757	0.833 ± 0.221	0.523
SPOT-RNAc	**0.736**	0.823	**0.777**	**0.777**	0.833 ± 0.249	

Note: The overall F1-score is harmonic mean of precision and recall for all RNA pairs. PPV denotes positive predictive value. MCC denotes Matthews’ correlation coefficient. The star * denotes the use of evolution information. Methods with an ending of “c” indicate the use of chain concatenation for RNA–RNA interaction prediction. Median F1 means the median F1 value of F1 values for single RNAs. The *P*-value of a given method was calculated by against the result from SPOT-RNAc and was calculated using Student’s t-test. Bold values highlight the best performance values.

**Figure 5.**
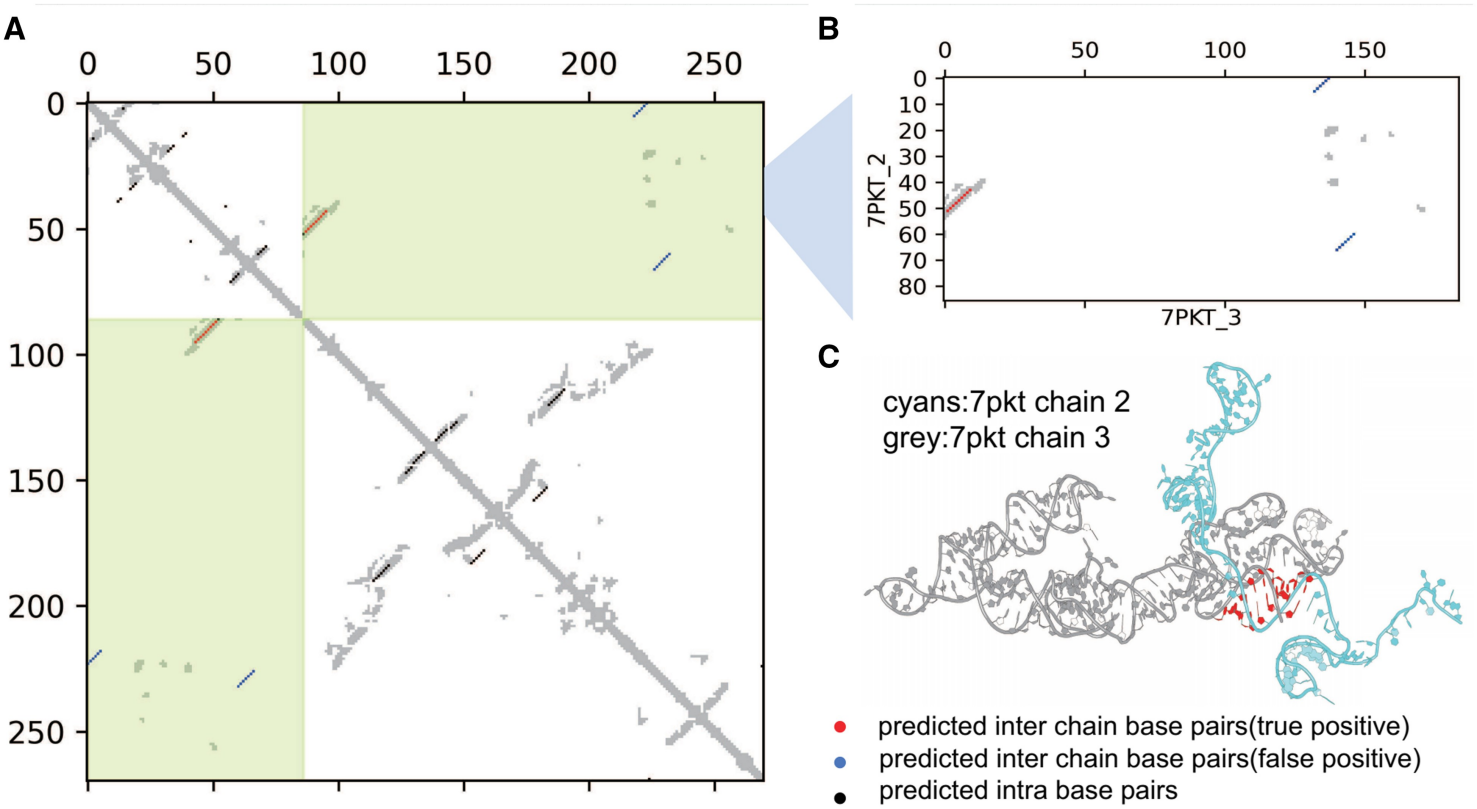
Accurate prediction of key inter-RNA base-pairing contacts in RNA complexes by SPOT-RNAc. Complex structure of large subunits of the *Chlamydomonas reinhardtii* mitoribosome (L2aRNA and L3bRNA in PDB ID 7PKT) with true distance-contact map and predicted intra and inter-RNA base pairs (A), inter-RNA base pairs only (B), and 3-D structure (C). Predicted intra-base pairs and inter-base pairs are denoted by black and red dots, respectively, in the base-pairing maps. In the 3-D structure (C), correctly predicted inter-RNA base pairs are highlighted in red.

We selected one example to illustrate SPOT-RNA’s performance in RRI prediction. Figure 4A displays predicted and actual base-pairing maps for the subunits L2a rRNA complexed with L3b rRNA from *Chlamydomonas reinhardtii* mitoribosome (PDB ID 7PKT, chain ID 2 and chain ID 3). Figure 5A displays the intra- and inter-base-pairing maps of these two RNAs with Fig. 5B for inter-base-pairing maps only. The F1-scores for intra-RNA base pairs are 0.32 for L2a rRNA and 0.30 for L3b rRNA, while the F1-score for inter-RNA base pairs is 0.556. Correctly predicted base pairs are highlighted with red dots in Fig. 5B and C, and their 3D locations were shown as red-colored bases in Fig. 5C. For this complex structure, precision for inter-RNA base pairs is 0.434, and sensitivity is relatively high (0.769).

## 4 Discussion

This study represents a comprehensive benchmark for assessing more than 20 methods in predicting RNA–RNA interactions. Previous benchmarks were constrained to interactions involving small RNAs without intra-RNA base pairs. For instance, Lai and Meyer (2016) compared 14 RRI methods based on experimentally confirmed interactions in fungal snoRNA–rRNA and bacterial sRNA–mRNA pairs. Umu and Gardner (2017) examined 15 RRI methods using a dataset focused on short linear base-pair matching. Antonov *et al.* (2019) compared 13 RRI methods on mammalian lncRNAs with experimentally proven hybridizations. In contrast, our study presents the first comprehensive benchmark of 23 RRI prediction methods using known RRI interactions derived from 3D structures at the base-pair level. Notably, most of these complexes (53/64) include RNAs with 3D structures and intra-RNA base pairs. Additionally, this study marks the first inclusion of deep-learning based methods for comparisons, utilizing chain concatenation.

In contrast to proteins, the available non-redundant data for RNA structures is limited. This raises concerns about the generalizability of deep-learning models trained on such limited data. Previous studies by Szikszai *et al.* (2022) and Qiu (2023) highlighted the challenges of deep-learning models when applied to unseen families not present in the training and validation sets. SPOT-RNA and SPOT-RNA2 were initially trained with a set of bpRNA dataset after removing all sequences in bpRNA with known 3D structures by CD-HIT (80% sequence identity cut-off), followed by transfer learning with 3D-structure-derived base pairs. To evaluate the adaptability of SPOT-RNA and SPOT-RNA2 beyond their training and validation data, we placed them to the test of untrained inter-RNA interactions. To avoid possible “over-trained” intra-RNA interactions affecting prediction of inter-RNA interactions, we excluded all single-chain structures in the complex test set with the structural similarity score (TM-score)≥0.3, compared to those in the training and validation sets of SPOT-RNA. Despite the above precaution, training/validation families were not entirely excluded because RNAs in the same family may not have an identical structure. Thus, we further established two family-excluded test sets (15 hetero-dimeric RNAs and 13 homo-dimeric RNAs). SPOT-RNA and SPOT-RNA2 remain best performance for inter and intra-RNA base-pair prediction, respectively, except for inter-base-pair prediction for homo-dimeric RNAs, for which no methods did well. The generalization capability of SPOT-RNA is further illustrated with its robust performance across different RNA types, even for RNAs with few intra-RNA structures (Supplementary Table S4). This result is also consistent with the top SPOT-RNA performance in a recent independent assessment of several secondary structure prediction techniques using self-cleavage ribozyme mutants (Qi *et al.* 2024).

The generalizability of SPOT-RNA is consistent with the fact that the folding of monomeric and complex structures of RNAs both are driven by the formation of Watson–Crick and non-canonical base pairs. SPOT-RNA and SPOT-RNA2 seem to have captured this general principle regardless of base pairs appeared in a single chain or between the chains. It should be noted that the main difference in base pairs of single-chain and those of complex prediction is the sequence distance between base pairs. The base pairs in dimeric structures are much more away from each other (nonlocal or long-distance) than those in monomeric structures when treating a dimer as a single chain. The result reported here indicates that the performance of SPOT-RNA can predict well those long-range contacts between chains.

SPOT-RNA2, which incorporates evolution and co-evolution information, outperforms SPOT-RNA for intra-RNA base pairs, aligning with previous findings (Table 3, Singh *et al.* 2021). However, SPOT-RNA2c underperforms SPOT-RNAc for inter-RNA base pairs. Notably, SPOT-RNA2 exhibits a positive correlation with the number of effective homologous sequences for intra-RNA base-pair prediction (PCC = 0.592, *P*-value = 1.47 × 10^–8^, Supplementary Fig. S1C), but this correlation nearly diminishes for SPOT-RNA2c for intra-RNA base pairs (PCC = 0.30, *P*-value = 0.03) and turned negative for inter-RNA base-pair prediction (PCC = −0.3, *P*-value = 0.02). This suggests that using linked sequences in SPOT-RNA2c for homology search may have provided harmful information for inferring inter-RNA interactions. In the future, it may be necessary to utilize sequences from the same species for homology searches, as co-evolution information can only be detected through inter-species comparisons via multiple sequence alignment (MSA) pairing as has been done for proteins (Bryant *et al.* 2022). However, the failure of SPOT-RNA2 in predicting inter-RNA interactions between homo-dimers indicates that the co-evolution signals between RNAs may be too weak to capture.

For predicting RNA–RNA interactions, we concatenated two chains (A and B) as a single chain. To eliminate artificial sequence-order dependence, we predicted results for both AB and BA chains and then calculated the average. Interestingly, we found that in some cases, one sequence order (e.g. BA) outperformed the other (i.e. AB). Upon closer examination, we discovered that for some RNA pairs, the order with a shorter separation in sequence positions for contacting base pairs tended to perform better. This observation is intuitively expected, as longer-range interactions are inherently more challenging to predict. However, there were some outliers deserving further studies.

It should be noted that RNA–RNA interactions between two monomerically structured RNAs occur likely between their exposed loops. Some of these loop–loop base pairs could become pseudoknots when two RNA chains are concatenated as a single chain. Among 4/23 methods investigated, only four (IntaRNA, UFold, SPOT-RNA, and SPOT-RNA2) can predict pseudoknots. We also found that like monomeric RNAs, these pseudoknots, although essential for RNA–RNA dimeric structure and functions, are only a small faction of all inter-RNA base pairs. In fact, among all RNA pairs investigated, only 8 out of 64 RNA interaction pairs in either A–B or B–A linkage contain pseudoknots. Moreover, the pseudoknots in 5/8 RRIs are made of lone (unstacked) base pair. The scarcity of these pseudoknots makes them extremely hard to predict (all four methods with near-zero F1-scores). Moreover, the small benchmark set makes the current study incapable of assessing if pseudoknot predictive capabilities can significantly improve the method performance. Nevertheless, previous studies on SPOT-RNA and SPOT-RNA2 demonstrated their excellent performance in pseudoknot prediction for monomeric RNAs (Singh *et al.* 2021).

## Supplementary Material

btaf289_Supplementary_Data

## Data Availability

The data underlying this article are available in https://github.com/meilanglang/RNA-RNA-Interaction.
