# Supplementary material for: Benchmarking the methods for predicting base pairs in RNA–RNA interactions
Source: Bioinformatics. 2025 May 6;41(6):btaf289. doi: 10.1093/bioinformatics/btaf289 (PMC12141194; doi:10.1093/bioinformatics/btaf289)
Supplement: btaf289_Supplementary_Data [file btaf289_supplementary_data.docx]

Supplementary Materials for

**Benchmarking the methods for predicting base pairs in RNA-RNA interactions**

Mei Lang^1^, Thomas Litfin^2^, Ke Chen^1^, Jian Zhan^1,3 *^ and Yaoqi Zhou^1,2*^

^1^Institute of Systems and Physical Biology, Shenzhen Bay Laboratory, Shenzhen, 518107, China

^2^Institute for Glycomics, Griffith University, Parklands Dr, Southport, QLD 4222, Australia

^3^Ribopeutic Inc, Guangzhou International Bio Island, Guangdong, 510320, China

*For correspondence, please email to Dr. Yaoqi Zhou ([zhouyq@szbl.ac.cn](mailto:zhouyq@szbl.ac.cn)) or Dr. Jian Zhan ([zhanjian@szbl.ac.cn](mailto:zhanjian@szbl.ac.cn))

Supplementary Figures and Tables


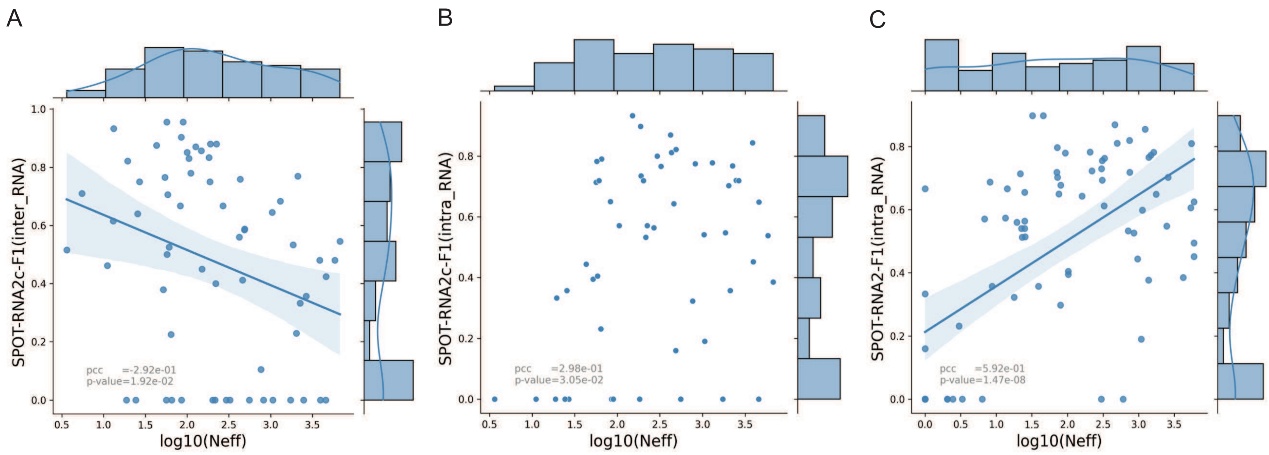


**Supplementary Figure S1.** (A) Inter-RNA F1-scores as function of the number of effective homologous sequences (Neff) given by SPOT-RNA2c. (B) Intra-RNA F1-scores (average F1-scores of two RNA complex) as a function of Neff given by SPOT-RNA2c. (C) intra-RNA F1-scores as a function of Neff given by SPOT-RNA2 with a positive correlation coefficient of 0.592.

**Supplementary Table S1:** List of PDB IDs of 64 RNA-RNA complexes in the test set**.**

#PDB_ID chain1_chain2

rRNA-containing (8)：7MQ8_L0_L2; 7PKT_2_3; 7PKT_3_6; 7PKT_3_4; 7PKT_4_5; 8A22_A2_A3; 8A22_A4_A5; 8A22_B1_B2.

snRNA/snoRNA/mRNA-containing and intra base pair >= 4 (40)：7D4I_3A_5A; 7D5S_3A_5A; 7D5T_3A_5A; 7D63_3A_5A; 7SUK_L0_L2; 7W59_G_F; 7W59_G_H; 7W59_F_H; 7W5A_F_G; 5GMK_L_M; 5LJ3_I_Z; 5LQW_2_9; 5MQF_2_6; 5NRL_2_6; 5NRL_2_I; 5NRL_4_6; 5WSG_E_L; 5Y88_D_E; 5Y88_E_F; 5ZWM_F_H; 5ZWM_F_I; 5ZWM_G_H; 6AH0_F_H; 6AH0_F_I; 6AHD_G_H; 6EXN_2_6; 6EXN_2_I; 6FF4_2_Z; 6J6G_B_L; 6J6N_B_L; 6QDV_2_6; 6QDV_2_I; 6QX9_6_2; 6QX9_6_4; 7ABI_6_2; 7B9V_2_6; 7DCO_F_H; 7DVQ_G_H; 7OQB_I_2; 5WSG_L_M.

Lacking intra-RNA interactions (16) :7V01_G_U; 7WAH_R_D; 7XSS_A_D; 7XT4_a_r; 7Y83_B_C; 7YNA_B_C; 5GIO_G_H; 6IFL_I_J; 6O1O_M_N; 6O7H_G_H; 6S91_U_V; 1DZ5_C_D; 7SBB_X_Z; 6NUD_H_U; 6ZYM_2_Y; 3SIV_C_F;

**Supplementary Table S2** The performance SPOT-RNAc concatenation with different linkers on the base pair level.

| Methods | PPV/ Precision | Sensitivity/ Recall | Overall F1-Score | MCC | P-value |
| --- | --- | --- | --- | --- | --- |
| SPOT-RNAc-GGG | 0.588 | 0.538 | 0.562 | 0.562 | 0.046 |
| SPOT-RNAc-UUU | 0.595 | 0.539 | 0.566 | 0.566 | 0.046 |
| SPOT-RNAc-CCC | 0.593 | 0.541 | 0.566 | 0.566 | 0.114 |
| SPOT-RNAc-AAA | **0.618** | 0.540 | 0.576 | **0.577** | 0.162 |
| SPOT-RNAc-NoLink | 0.606 | **0.548** | **0.576** | 0.576 |  |

Note: The P-value of a given method was calculated by comparing against the result from SPOT-RNAc-NoLink and was calculated using Student's t-test.

**Supplementary Table S3:** Performance Comparison of 23 Predictors of Inter-RNA Base Pairs on 64 Complexes of RNA Structures Unseen by SPOT-RNA and SPOT-RNA2 by ignoring the existence of pseudoknots in performance evaluation [Eight complexes* have pseudoknots in AB or BA linkage. They are simply ignored during the performance evaluation].

| Methods | | PPV/ Precision | Sensitivity/Recall | Overall F1-Score | MCC | Individual F1-Score Median ± Std | P-value |
| --- | --- | --- | --- | --- | --- | --- | --- |
| GUUGle | | 0.159 | 0.108 | 0.129 | 0.130 | 0.000 ± 0.241 | 6.00e-15 |
| RIsearch | | 0.176 | 0.260 | 0.210 | 0.213 | 0.000 ± 0.319 | 1.56e-12 |
| RNAaliduplex | | 0.484 | 0.152 | 0.231 | 0.271 | 0.000 ± 0.296 | 1.40e-11 |
| RNAplex-cA | | 0.477 | 0.152 | 0.231 | 0.269 | 0.000 ± 0.296 | 1.40e-11 |
| DuplexFold | | 0.182 | 0.458 | 0.260 | 0.287 | 0.200 ± 0.344 | 4.46e-08 |
| RNAplex-c | | 0.189 | 0.455 | 0.267 | 0.292 | 0.216 ± 0.343 | 1.44e-08 |
| RNAduplex | | 0.195 | 0.505 | 0.281 | 0.313 | 0.229 ± 0.329 | 1.32e-08 |
| RNAup | | 0.281 | 0.367 | 0.318 | 0.320 | 0.000 ± 0.291 | 9.54e-13 |
| RNAplex-a | | 0.364 | 0.293 | 0.325 | 0.326 | 0.000 ± 0.331 | 1.89e-09 |
| AccessFold | | 0.317 | 0.504 | 0.389 | 0.398 | 0.349 ± 0.315 | 3.28e-06 |
| PETcoFold | | 0.433 | 0.375 | 0.402 | 0.402 | 0.377 ± 0.352 | 5.66e-05 |
| RNAfoldc | | 0.378 | 0.587 | 0.460 | 0.470 | 0.441 ± 0.318 | 3.20e-03 |
| NUPACK | | 0.439 | 0.486 | 0.461 | 0.461 | 0.467 ± 0.355 | 1.05e-03 |
| MXfold2c | | 0.385 | 0.608 | 0.471 | 0.483 | 0.448 ± 0.321 | 1.29e-03 |
| bifold | | 0.409 | 0.563 | 0.474 | 0.479 | 0.454 ± 0.325 | 0.008 |
| PairFold | | 0.421 | 0.567 | 0.483 | 0.488 | 0.460 ± 0.339 | 3.72e-03 |
| RNAmultifold | | 0.417 | 0.588 | 0.488 | 0.494 | 0.486 ± 0.326 | 0.007 |
| RNAcoFold | | 0.419 | 0.588 | 0.489 | 0.495 | 0.486 ± 0.326 | 0.007 |
| IntaRNA2.0 | 0.540 | | 0.449 | 0.490 | 0.492 | 0.405 ± 0.378 | 4.15e-05 |
| EternaFoldc | 0.433 | | **0.626** | 0.512 | 0.520 | 0.483 ± 0.315 | 0.044 |
| UFoldc | 0.499 | | 0.538 | 0.518 | 0.517 | 0.536 ± 0.314 | 0.020 |
| SPOT-RNA2c | 0.570 | | 0.546 | 0.558 | 0.557 | 0.535 ± 0.331 | 3.20e-03 |
| SPOT-RNAc | **0.650** | | 0.557 | **0.600** | **0.601** | **0.630** ± 0.328 |  |

*PDB ID with pseudoknot pairs: 7PKT_23;7PKT_45; 8A22_A4A5; 8A22_B1B2; 5LJ3_IZ;5WSG_EL;6QX9_64; and 6ZYM_2Y

**Supplementary Table S4** Performance comparison of 10 predictors (Top 10 predictor on 64 complexes) of inter-RNA base pairs on different RNA categories.

| Methods | PPV/ Precision | Sensitivity/ Recall | Overall F1-Score | MCC | Individual F1-Score Median ± Std | | P-value |
| --- | --- | --- | --- | --- | --- | --- | --- |
| **rRNA-containing (N=8)** | | | | | | | |
| IntaRNA2.0 | 0.188 | 0.121 | 0.147 | 0.150 | 0.000 ± 0.237 | | 0.012 |
| MXfold2c | 0.169 | 0.318 | 0.221 | 0.231 | 0.245 ± 0.212 | | 0.030 |
| bifold | 0.190 | 0.306 | 0.234 | 0.240 | 0.239 ± 0.239 | | 0.072 |
| PairFold | 0.204 | 0.299 | 0.243 | 0.247 | 0.202 ± 0.217 | | 0.029 |
| RNAmultifold | 0.195 | 0.344 | 0.249 | 0.258 | 0.245 ± 0.242 | | 0.081 |
| RNAcoFold | 0.195 | 0.344 | 0.249 | 0.258 | 0.245 ± 0.242 | | 0.081 |
| UFoldc | 0.196 | **0.382** | 0.259 | 0.273 | 0.281 ± 0.172 | | 0.075 |
| EternaFoldc | 0.220 | 0.357 | 0.272 | 0.28 | 0.277 ± 0.203 | | 0.050 |
| SPOT-RNA2c | 0.297 | 0.331 | 0.313 | 0.313 | 0.281 ± 0.234 | | 0.237 |
| SPOT-RNAc | **0.372** | 0.369 | **0.370** | **0.370** | **0.347** ± 0.188 | |  |
| **snRNA/snoRNA/mRNA-containing and intra base pair >= 4 (N=40)** | | | | | | | |
| UFoldc | 0.376 | 0.437 | 0.404 | 0.405 | 0.405 ± 0.273 | 0.024 | |
| bifold | 0.351 | 0.495 | 0.411 | 0.416 | 0.386 ± 0.299 | 0.075 | |
| PairFold | 0.358 | 0.489 | 0.413 | 0.418 | 0.337 ± 0.321 | 0.054 | |
| IntaRNA2.0 | 0.476 | 0.367 | 0.414 | 0.417 | 0.331 ± 0.354 | 4.07e-3 | |
| MXfold2c | 0.337 | **0.546** | 0.417 | 0.428 | 0.402 ± 0.292 | 0.043 | |
| RNAmultifold | 0.371 | 0.514 | 0.431 | 0.436 | 0.464 ± 0.304 | 0.090 | |
| RNAcoFold | 0.373 | 0.514 | 0.432 | 0.437 | 0.464 ± 0.304 | 0.090 | |
| EternaFoldc | 0.384 | 0.567 | 0.458 | 0.466 | 0.474 ± 0.293 | 0.295 | |
| SPOT-RNA2c | 0.578 | 0.436 | 0.497 | 0.501 | 0.480 ± 0.319 | 0.051 | |
| SPOT-RNAc | **0.588** | 0.448 | **0.509** | **0.513** | **0.534** ± 0.325 |  | |
| **lacking intra-RNA interactions (N=16)** | | | | | | | |
| UFoldc | 0.678 | 0.836 | 0.749 | 0.747 | 0.817 ± 0.242 | | 0.056 |
| IntaRNA2.0 | 0.755 | 0.748 | 0.751 | 0.746 | 0.839 ± 0.346 | | 0.091 |
| SPOT-RNA2c | 0.678 | 0.849 | 0.754 | 0.753 | 0.827 ± 0.243 | | 0.037 |
| bifold | 0.699 | 0.830 | 0.759 | 0.757 | 0.839 ± 0.246 | | 0.143 |
| RNAmultifold | 0.705 | 0.830 | 0.762 | 0.760 | 0.839 ± 0.243 | | 0.084 |
| RNAcoFold | 0.705 | 0.830 | 0.762 | 0.760 | 0.839 ± 0.243 | | 0.084 |
| MXfold2c | 0.693 | **0.855** | 0.766 | 0.765 | 0.829 ± 0.242 | | 0.066 |
| PairFold | 0.723 | 0.830 | 0.773 | 0.769 | 0.839 ± 0.241 | | 0.191 |
| EternaFoldc | 0.720 | 0.852 | 0.780 | 0.778 | 0.842 ± 0.256 | | 0.181 |
| SPOT-RNAc | **0.732** | 0.842 | **0.783** | **0.780** | **0.837** ± 0.238 | |  |

Notes: The overall F1-score is harmonic mean of precision and recall for all RNA pairs. PPV denotes positive predictive value. MCC denotes Matthew’s correlation coefficient. The star * denotes the use of evolution information**.** Methods with an ending of “c” indicate the use of chain concatenation for RNA-RNA interaction prediction. Median F1 means the median F1 value of single RNA. The P-value of a given method was computed by against the result from SPOT-RNAc and was calculated using Student's t-test. rRNA, snRNA, snoRNA, mRNA pairs indicate that an RNA complex with at least one chain is rRNA, snRNA, snoRNA or mRNA and intra_RNA base pair number be equal or greater than 4. Lacking intra-RNA interactions indicates the chain with less than 4 intra base pair.

**Supplementary Table S5** Performance comparison of all predictors on 15 hetero-dimer complexes of intra-RNA base pairs that are not in RNA families containing in TR0/VL0 (bpRNA) and TR1/VL1 for training SPOT-RNA and SPOT-RNA2.

| Methods | PPV/ Precision | Sensitivity/Recall | Overall F1-Score | MCC | Individual F1-Score Median ± Std | P-value |
| --- | --- | --- | --- | --- | --- | --- |
| PETcoFold | 0.114 | 0.040 | 0.059 | 0.064 | 0.000 ± 0.136 | 0.031 |
| EternaFoldc | 0.125 | 0.139 | 0.132 | 0.126 | 0.000 ± 0.201 | 0.059 |
| PairFold | 0.365 | 0.418 | 0.390 | 0.387 | 0.000 ± 0.217 | 0.036 |
| UFoldc | 0.662 | 0.448 | 0.534 | 0.542 | 0.000 ± 0.290 | 0.113 |
| SPOT-RNAc | **0.735** | 0.428 | 0.541 | 0.559 | 0.000 ± 0.322 | 0.375 |
| MXfold2c | 0.650 | 0.463 | 0.541 | 0.546 | 0.000 ± 0.291 | 0.122 |
| SPOT-RNA | 0.689 | 0.453 | 0.547 | 0.557 | 0.000 ± 0.323 | 0.387 |
| SPOT-RNA2c | 0.532 | **0.622** | 0.573 | 0.572 | **0.333** ± 0.299 | 0.696 |
| RNAfoldc | 0.646 | 0.527 | 0.580 | 0.582 | 0.000 ± 0.301 | 0.057 |
| RNAcoFold | 0.694 | 0.507 | 0.586 | 0.591 | 0.000 ± 0.304 | 0.072 |
| RNAmultifold | 0.694 | 0.507 | 0.586 | **0.591** | 0.000 ± 0.304 | 0.072 |
| SPOT-RNA2 | 0.576 | 0.602 | **0.589** | 0.586 | 0.000 ± 0.333 |  |

Note: The overall F1-score is harmonic mean of precision and recall for all RNA pairs. PPV denotes positive predictive value. MCC denotes Matthews’ correlation coefficient. The star * indicates the use of evolution information. Std means standard deviation**.** Median F1 means the median F1 value of individual RNA chains. The P-value of a given method is calculated by against the result of SPOT-RNA2 and was calculated using Student's t-test.

**Supplementary Table S6** Performance comparison of all predictors on 13 homo-dimer complexes of inter-RNA base pairs that are not in RNA families containing in TR0/VL0 (bpRNA) and TR1/VL1 for training SPOT-RNA and SPOT-RNA2.

| Methods | PPV/ Precision | Sensitivity/Recall | Overall F1-Score | MCC | Individual F1-Score Median ± Std | P-value |
| --- | --- | --- | --- | --- | --- | --- |
| NUPACK | 0.059 | 0.129 | 0.081 | 0.084 | 0.000 ± 0.281 | 0.182 |
| RIsearch | 0.021 | 0.105 | 0.035 | 0.042 | 0.000 ± 0.171 | 0.036 |
| RNAaliduplex | 0.032 | 0.058 | 0.041 | 0.041 | 0.000 ± 0.178 | 0.031 |
| RNAplex-cA | 0.051 | 0.058 | 0.054 | 0.053 | 0.000 ± 0.178 | 0.031 |
| RNAfoldc | 0.064 | 0.129 | 0.086 | 0.088 | 0.000 ± 0.205 | 0.054 |
| bifold | 0.077 | 0.152 | 0.102 | 0.106 | 0.000 ± 0.278 | 0.213 |
| PairFold | 0.070 | 0.211 | 0.105 | 0.118 | 0.000 ± 0.266 | 0.234 |
| MXfold2c | 0.091 | 0.216 | 0.128 | 0.138 | 0.000 ± 0.287 | 0.189 |
| RNAup | 0.125 | 0.152 | 0.137 | 0.136 | 0.000 ± 0.346 | 0.264 |
| RNAmultifold | 0.101 | 0.211 | 0.137 | 0.143 | 0.000 ± 0.309 | 0.309 |
| RNAcoFold | 0.101 | 0.211 | 0.137 | 0.143 | 0.000 ± 0.309 | 0.309 |
| RNAduplex | 0.097 | 0.281 | 0.144 | 0.162 | 0.000 ± 0.300 | 0.328 |
| RNAplex-c | 0.097 | 0.281 | 0.144 | 0.162 | 0.000 ± 0.293 | 0.313 |
| IntaRNA2.0 | 0.176 | 0.129 | 0.149 | 0.149 | 0.000 ± 0.288 | 0.048 |
| PETcoFold | 0.300 | 0.105 | 0.156 | 0.177 | 0.000 ± 0.161 | 0.028 |
| RNAplex-a | 0.165 | 0.158 | 0.161 | 0.159 | 0.000 ± 0.252 | 0.129 |
| SPOT-RNA2c | 0.116 | 0.269 | 0.162 | 0.174 | 0.000 ± 0.313 | 0.320 |
| DuplexFold | 0.109 | 0.339 | 0.165 | 0.189 | 0.000 ± 0.295 | 0.381 |
| SPOT-RNAc | 0.151 | 0.287 | 0.198 | 0.206 | 0.000 ± 0.310 | 0.334 |
| EternaFoldc | 0.168 | 0.275 | 0.209 | 0.213 | 0.000 ± 0.318 | 0.367 |
| UFoldc | 0.165 | 0.380 | 0.230 | 0.248 | 0.175 ± 0.281 | 0.460 |
| GUUGle | 0.351 | 0.269 | 0.305 | 0.306 | 0.000 ± 0.369 | 0.527 |
| AccessFold | **0.312** | **0.456** | **0.370** | **0.376** | **0.246** ± 0.290 |  |

Note:The P-value of a given method was calculated by against the result from AccessFold and was calculated using Student's t-test.

**Supplementary Table S7** Performance comparison of all predictors on 13 homodimers of intra-RNA base pairs that are not in RNA families containing in TR0/VL0 (bpRNA) and TR1/VL1 for training SPOT-RNA and SPOT-RNA2.

| Methods | PPV/ Precision | Sensitivity/Recall | Overall F1-Score | MCC | Individual F1-Score Median ± Std | P-value |
| --- | --- | --- | --- | --- | --- | --- |
| PETcoFold | **0.854** | 0.200 | 0.324 | 0.411 | 0.000 ± 0.333 | 1.44e-5 |
| MXfold2c | 0.601 | 0.286 | 0.388 | 0.411 | 0.159 ± 0.296 | 7.55e-5 |
| SPOT-RNAc | 0.820 | 0.273 | 0.410 | 0.471 | 0.053 ± 0.278 | 1.34e-5 |
| PairFold | 0.711 | 0.295 | 0.417 | 0.456 | 0.289 ± 0.322 | 1.51e-4 |
| UFoldc | 0.667 | 0.351 | 0.460 | 0.481 | 0.346 ± 0.314 | 1.60e-4 |
| RNAcoFold | 0.821 | 0.328 | 0.469 | 0.517 | 0.188 ± 0.323 | 2.83e-4 |
| RNAmultifold | 0.821 | 0.328 | 0.469 | 0.517 | 0.188 ± 0.323 | 2.83e-4 |
| RNAfoldc | 0.643 | 0.401 | 0.494 | 0.505 | 0.244 ± 0.364 | 8.83e-4 |
| SPOT-RNA2c | 0.690 | 0.404 | 0.510 | 0.525 | 0.450 ± 0.308 | 4.19e-4 |
| EternaFoldc | 0.746 | 0.424 | 0.541 | 0.560 | 0.503 ± 0.348 | 1.93e-3 |
| SPOT-RNA | 0.620 | 0.503 | 0.555 | 0.556 | 0.435 ± 0.306 | 5.77e-4 |
| SPOT-RNA2 | 0.717 | **0.732** | **0.724** | **0.722** | **0.686** ± 0.367 |  |

Note:The P-value of a given method was calculated by against the result from AccessFold and was calculated using Student's t-test.

**Supplementary Table S8:** List of PDB IDs of 15 RNA-RNA hetero-dimers in the test set that not match to Rfam family of training data of SPOT-RNA and SPOT-RNA2**.**

#PDB_ID chain1_chain2

7SBB_X_Z ; 7V01_G_U; 7WAH_R_D; 7XSS_A_D; 7XT4_a_r; 7Y83_B_C; 7YNA_B_C; 6IFL_I_J; 6NUD_H_U; 6O1O_M_N; 6O7H_G_H; 6S91_U_V; 6G7Z_A_B; 8I3Z_A_B; 8T2P_A_B;

**Supplementary Table S9:** List of PDB IDs of 13 RNA-RNA homo-dimer in the test set that not match to Rfam family of training data of SPOT-RNA and SPOT-RNA2.

2IL9_A_M; 2L1F_A_B; 7KGA_A_B; 2ADT_A_B; 6V9D_B_E; 6ZDU_C_D; 1DZ5_C_D; 6JQ5_A_B; 7JJU_A_B; 7JRR_A_A-2; 7JRS_A_B; 7JRT_A_B; 5GIO_G_H.

Supplementary Method Description

1. Tools Settings

For some tools setting, we followed the excellent review by Lai and Meyer(Lai and Meyer, 2016). But due to the different sequence length of our benchmark data set, we changed some parameters.

- 1. Interaction only prediction tools

Inputs (target.fa and query.fa) are two standard FASTA ﬁles of two RNA sequences. Each file contains one header line followed by an RNA sequence.

- - 1. RNAduplex

RNAduplex(Lorenz *et al.*, 2011) designed for computing the structure upon hybridization of two RNA strands. Algorithmically, it is a modiﬁcation of Zuker’s classical RNA secondary structure algorithm, simplified to just consider intermolecular base pairs.

RNAduplex is a part of the ViennaRNA Package 2.5(Lorenz *et al.*, 2011), we downloaded the package from <https://www.tbi.univie.ac.at/RNA/#download.>

cat query.fa target.fa | RNAduplex -s > output.txt

-s sort the printed output by free energy

- - 1. RNAplex-c

RNAplex-c(Tafer and Hofacker, 2008) was especially designed to quickly find possible hybridization sites for a query RNA in large RNA databases. It utilizes a slightly different energy model which reduces the computational time significantly, compared to RNAhybrid.

RNAplex-c is also a part of the ViennaRNA Package 2.5(Lorenz *et al.*, 2011) and the package was obtained from <https://www.tbi.univie.ac.at/RNA/#download>.

RNAplex -q query.fa -t target.fa > output.txt

- - 1. [DuplexFold](https://rna.urmc.rochester.edu/RNAstructureWeb/Servers/DuplexFold/DuplexFold.html)

DuplexFold(Reuter and Mathews, 2010) predicts the lowest free energy structure for two interacting sequences, not allowing intramolecular base pairs.

DuplexFold is a part of the RNAstructure Package 6.4(Reuter and Mathews, 2010) and we obtained the package from <https://rna.urmc.rochester.edu/RNAstructure.html.>

DuplexFold query.fa target.fa > output.txt

- - 1. RIsearch

 RIsearch(Wenzel *et al.*, 2012) enables quick localization of potential near-complementary interactions between given query and target sequences. RIsearch uses a modified Smith-Waterman-Gotoh algorithm based on di-nucleotides to approximate nearest-neighbor energy parameters.

We obtained the tool from <https://rth.dk/resources/risearch/.>

RIsearch -q query.fa -t target.fa -p > output.txt

-p report predictions in detailed format

- - 1. RNAplex-cA

An updated version of RNAplex(Tafer and Hofacker, 2008) can additionally take alignments as input, and like RNAaliduplex, also incorporates a covariation score into the stability of the predicted duplex in the style of RNAalifold. We also refer to this as RNAplex-cA as the review paper did(Lai and Meyer, 2016).

RNAplex-cA is also part of the ViennaRNA Package 2.5(Lorenz *et al.*, 2011) and the package was obtained from <https://www.tbi.univie.ac.at/RNA/#download>.

RNAplex -q query_alignment.fa -t target_alignment.fa -A > output.txt

-A: Instructs the tool to compute interactions based on alignments

- - 1. RNAaliduplex

RNAaliduplex predicts conserved RNA-RNA interactions between two alignments, also part of the same ViennaRNA Package 2.5(Lorenz *et al.*, 2011). The calculation takes only inter−molecular base pairs into account. The use of alignments allows one to focus on binding sites that are evolutionary conserved. The program must read two alignments of RNA sequences in CLUSTAL format and predicts optimal and suboptimal binding sites, hybridization energies, and the corresponding structures. RNAaliduplex also is a part of ViennaRNA Package 2.5(Lorenz *et al.*, 2011). We downloaded the package from <https://www.tbi.univie.ac.at/RNA/#download.>

RNAaliduplex query_alignment.aln target_alignment.aln > output.txt

- - 1. GUUGle

GUUGle(Gerlach and Giegerich, 2006)Version 1.2 obtained from https://bibiserv2.cebitec.uni-bielefeld.de/guugle?id=guugle_view_ download

guugle -d 5 target.fa query.fa > output.txt

-d Obligatory option, minimum interaction length to be output

- 1. Accessibility prediction tools
     1. IntaRNA

IntaRNA2.0(Mann *et al.*, 2017) is a program for fast and accurate prediction of interactions between two RNA molecules. It was designed to predict mRNA target sites for given non-coding RNAs (ncRNAs) like eukaryotic microRNAs (miRNAs) or bacterial small RNAs (sRNAs), but it can also be used for predicting other types of RNA-RNA interactions.

Version 2.3.1 download from http://www.bioinf.uni-freiburg.de/Software/#IntaRNA

IntaRNA -q query.fa -t target.fa --outMode=C --outOverlap=N -n 1 > output.txt

--outMode=C 'C' CSV output (see --outCsvCols)

--outOverlap=N suboptimal output : interactions can overlap, 'N' in none of the sequences

-n number of (sub)optimal interactions to report

- - 1. RNAup

RNAup(Mückstein *et al.*, 2006) calculates the thermodynamics of RNA−RNA interactions, by decomposing the binding into two stages: (1) the probability that a potential binding sites remains unpaired (equivalent to the free energy needed to open the site) is computed; (2) this accessibility is combined with the interaction energy to obtain the total binding energy. All calculations are done by computing partition functions over all possible conformations.

RNAup is also a part of the ViennaRNA Package 2.5(Lorenz *et al.*, 2011) . The package was obtained from <https://www.tbi.univie.ac.at/RNA/#download>.

cat query.fa target.fa | RNAup -b --interaction_pairwise > output.txt

--interaction_pairwise: Activate the pairwise interaction mode.

- - 1. RNAplex-a

The new version of RNAplex-c (Tafer and Hofacker, 2008), supplies a new option -a, which can take in RNAplfold.

And we also named it as RNAplex-a. RNAplex-a and RNAplfold are a part of ViennaRNA Package 2.5(Lorenz *et al.*, 2011). We downloaded the package from <https://www.tbi.univie.ac.at/RNA/#download.>

cat query.fa target.fa | RNAplfold -O -u min[seq1,seq2] -W 10

RNAplex -q query.fa -t target.fa -a accessibility_dir -a -l min_seq_len > output.txt

-O Switch output from probabilities to their logarithms

-u Compute the mean probability that regions of length 1 to a given length are unpaired. min_seq_len is minimum rounded known interaction length.

-W Average the pair probabilities over the window of a given size.

-l Maximal length of interaction. min_seq_len is minimum rounded known interaction length.

- 1. DeepLearning models based concatenation tools

Inputs target_seq and query_seq as a linked single RNA sequence.

- - 1. SPOT-RNA

SPOT-RNA(Singh *et al.*, 2019) is an end-to-end predictor of intra-RNA base pairs by deep learning. The method was initially trained from a large approximate database of RNA secondary structures (bpRNA(Danaee *et al.*, 2018)), followed by transfer learning on more precise base pairs from Protein Data Bank RNA 3D structures. SPOT-RNA employs ResNet combined with a 2D-BLSTM as the main network architecture and takes single sequence information as it’s input feature. SPOT-RNA was initially trained on the bpRNA dataset and then fine-tuned on the structured RNA from the PDB dataset. For more detail information can be found in the original paper.

We download SPOT-RNA from <https://github.com/jaswindersingh2/SPOT-RNA/.>

SPOT-RNA -i qurey_seq + target_seq

- - 1. SPOT-RNA2

SPOT-RNA(Singh *et al.*, 2021) was subsequently improved by SPOT-RNA2, which incorporated evolution profiles and mutational coupling automatically generated by RNAcmap(Zhang *et al.*, 2021). Except for the input feature, the training data set and the deep learning network architecture is the same as SPOT-RNA.

We download SPOT-RNA2 from [https://github.com/jaswindersingh2/SPOT-RNA2/.](https://github.com/jaswindersingh2/SPOT-RNA/.)

SPOT-RNA2 -i qurey_seq + target_seq

- - 1. UFold

UFold(Fu *et al.*, 2022) is a RNA secondary structure prediction tool by using deep learning.

We download UFold from https: //github.com/uci-cbcl/UFold.

predict.py qurey_seq + target_seq -nc true

- - 1. MXfold2

MXfold2(Sato *et al.*, 2021) is a RNA secondary structure prediction tool by using deep learning. We download it from <https://github.com/keio-bioinformatics/>

- 1. MFE/partition-based concatenation tools

Input target_seq and query_seq as a linked single RNA sequence.

- - 1. RNAfold

RNAfold(Andronescu *et al.*, 2005) calculates minimum free energy secondary structures and partition function of RNAs. Here, we also employed it to predict intermolecular base pairs by chain concatenation.

RNAfold is a part of ViennaRNA Package 2.5(Lorenz *et al.*, 2011). We downloaded the package from <https://www.tbi.univie.ac.at/RNA/#download.>

RNAfold --MAE qurey_seq + target_seq

- - 1. RNAcoFold

RNAcofold(Bernhart *et al.*, 2006) calculates secondary structures of two RNAs with dimerization. The program works much like RNAfold, But allows one to specify two RNA sequences which are then allowed to form a dimer structure.

RNAcoFold is part of ViennaRNA Package 2.5(Lorenz *et al.*, 2011). We downloaded the package from <https://www.tbi.univie.ac.at/RNA/#download.>

qurey_seq + “\’&\’” + target_seq | RNAcoFold > output.txt

- - 1. RNAmultifold

RNAmultifold(Lorenz *et al.*, 2011) computes secondary structures of multiple interacting RNAs. RNAmultifold is the successor of the RNA-RNA dimer interaction prediction tool RNAcofold and effectively lifts the restriction to just two interacting strands. It follows the same principle of concatenating the RNA strands that shall form a complex and then predicts MFE and partition function. Along with that, it can also compute equilibrium concentrations of the complexes formed.

RNAmultifold is part of ViennaRNA Package 2.5(Lorenz *et al.*, 2011). We downloaded the package from <https://www.tbi.univie.ac.at/RNA/#download.>

qurey_seq + “\’&\’” + target_seq | RNAmultifold > output.txt

- - 1. NUPAC

NUPAC(Dirks *et al.*, 2007) algorithms operate over two fundamental ensembles:

Complex ensemble: the ensemble of all (unpseudoknotted connected) secondary structures for an arbitrary number of interacting RNA or DNA strands.

Test tube ensemble: the ensemble of a dilute solution containing an arbitrary number of RNA or DNA strand species (introduced at user-specified concentrations) interacting to form an arbitrary number of complex species.

The version 4.0 user guide was from <https://docs.nupack.org/>

- - 1. PairFold

PairFold(Andronescu *et al.*, 2005) predicts the minimum free energy of secondary structure formed by two input DNA or RNA molecules.

We downloaded the tools from <http://www.rnasoft.ca/>

PairFold qurey_seq target_seq > output.txt

- - 1. AccessFold

AccessFold(DiChiacchio *et al.*, 2016) predicts bimolecular base pairs while accounting for the preexisting structure. AccessFold employs Pseudo-energy minimization to minimize the sum of free energy change and a pseudo-free energy penalty for bimolecular pairing of nucleotides that are unlikely to be accessible for bimolecular structure.

AccessFold is part of the RNAstructure Package 6.4(Reuter and Mathews, 2010). We obtained the package from <https://rna.urmc.rochester.edu/RNAstructure.html.>

AccessFold query.fa taget.fa > output.txt

- - 1. EternaFold

EternFold(Wayment-Steele *et al.*, 2022) is employed to predict secondary structure. We download package from https://www.github.com/eternagame/ EternaBench.

We run EternaFold with this command: "./src/contrafold predict - qurey_seq + target_seq -params parameters/EternaFoldParams.v1

- 1. complex joint
     1. bifold

bifold(Reuter and Mathews, 2010) predicts the lowest free energy structure for two interacting sequences, allowing intramolecular base pairs.

bifold is part of the RNAstructure Package 6.4(Reuter and Mathews, 2010). We obtained the package from <https://rna.urmc.rochester.edu/RNAstructure.html.>

bifold query.fa taget.fa > output.txt

- - 1. PETcoFold

PETcoFold(Seemann *et al.*, 2011) predicts the joint secondary structure including RNA-RNA interactions from two RNA alignments. Version 3.3 was obtained from http://rth.dk/resources/petcofold/download.php

PETcofold -f query_alignment.fa -f target_alignment.fa --intermol > output.txt

--intermol Structure output of intermolecular base pairs

### Reference

Andronescu,M. et al. (2005) Secondary Structure Prediction of Interacting RNA Molecules. Journal of Molecular Biology, 345, 987–1001.

Bernhart,S.H. et al. (2006) Partition function and base pairing probabilities of RNA heterodimers. Algorithms Mol Biol, 1, 3.

Danaee,P. et al. (2018) bpRNA: large-scale automated annotation and analysis of RNA secondary structure. Nucleic Acids Res, 46, 5381–5394.

DiChiacchio,L. et al. (2016) AccessFold: predicting RNA-RNA interactions with consideration for competing self-structure. Bioinformatics, 32, 1033–1039.

Dirks,R.M. et al. (2007) Thermodynamic Analysis of Interacting Nucleic Acid Strands. SIAM Rev., 49, 65–88.

Fu,L. et al. (2022) UFold: fast and accurate RNA secondary structure prediction with deep learning. Nucleic Acids Res, 50, e14.

Gerlach,W. and Giegerich,R. (2006) GUUGle: a utility for fast exact matching under RNA complementary rules including G-U base pairing. Bioinformatics, 22, 762–764.

Lai,D. and Meyer,I.M. (2016) A comprehensive comparison of general RNA–RNA interaction prediction methods. Nucleic Acids Res, 44, e61.

Lorenz,R. et al. (2011) ViennaRNA Package 2.0. Algorithms Mol Biol, 6, 26.

Mann,M. et al. (2017) IntaRNA 2.0: enhanced and customizable prediction of RNA–RNA interactions. Nucleic Acids Research, 45, W435–W439.

Mückstein,U. et al. (2006) Thermodynamics of RNA–RNA binding. Bioinformatics, 22, 1177–1182.

Reuter,J.S. and Mathews,D.H. (2010) RNAstructure: software for RNA secondary structure prediction and analysis. BMC Bioinformatics, 11, 129.

Sato,K. et al. (2021) RNA secondary structure prediction using deep learning with thermodynamic integration. Nat Commun, 12, 941.

Seemann,S.E. et al. (2011) PETcofold: predicting conserved interactions and structures of two multiple alignments of RNA sequences. Bioinformatics, 27, 211–219.

Singh,J. et al. (2019) RNA secondary structure prediction using an ensemble of two-dimensional deep neural networks and transfer learning. Nat Commun, 10, 5407.

Singh,Jaswinder et al. (2021) Improved RNA secondary structure and tertiary base-pairing prediction using evolutionary profile, mutational coupling and two-dimensional transfer learning. Bioinformatics, 37, 2589–2600.

Tafer,H. and Hofacker,I.L. (2008) RNAplex: a fast tool for RNA-RNA interaction search. Bioinformatics, 24, 2657–2663.

Wayment-Steele,H.K. et al. (2022) RNA secondary structure packages evaluated and improved by high-throughput experiments. Nat Methods, 19, 1234–1242.

Wenzel,A. et al. (2012) RIsearch: fast RNA–RNA interaction search using a simplified nearest-neighbor energy model. Bioinformatics, 28, 2738–2746.

Zhang,T. et al. (2021) RNAcmap: a fully automatic pipeline for predicting contact maps of RNAs by evolutionary coupling analysis. Bioinformatics, 37, 3494–3500.
